# Supplementary material for: Global meta-analysis reveals overall higher nocturnal than diurnal activity in insect communities
Source: Nat Commun. 2024 Apr 15;15:3236. doi: 10.1038/s41467-024-47645-2 (PMC11018786; doi:10.1038/s41467-024-47645-2)
Supplement: Supplementary file 1 — Supplementary Information [file 41467_2024_47645_MOESM1_ESM.pdf]

Supplementary Figures and Tables

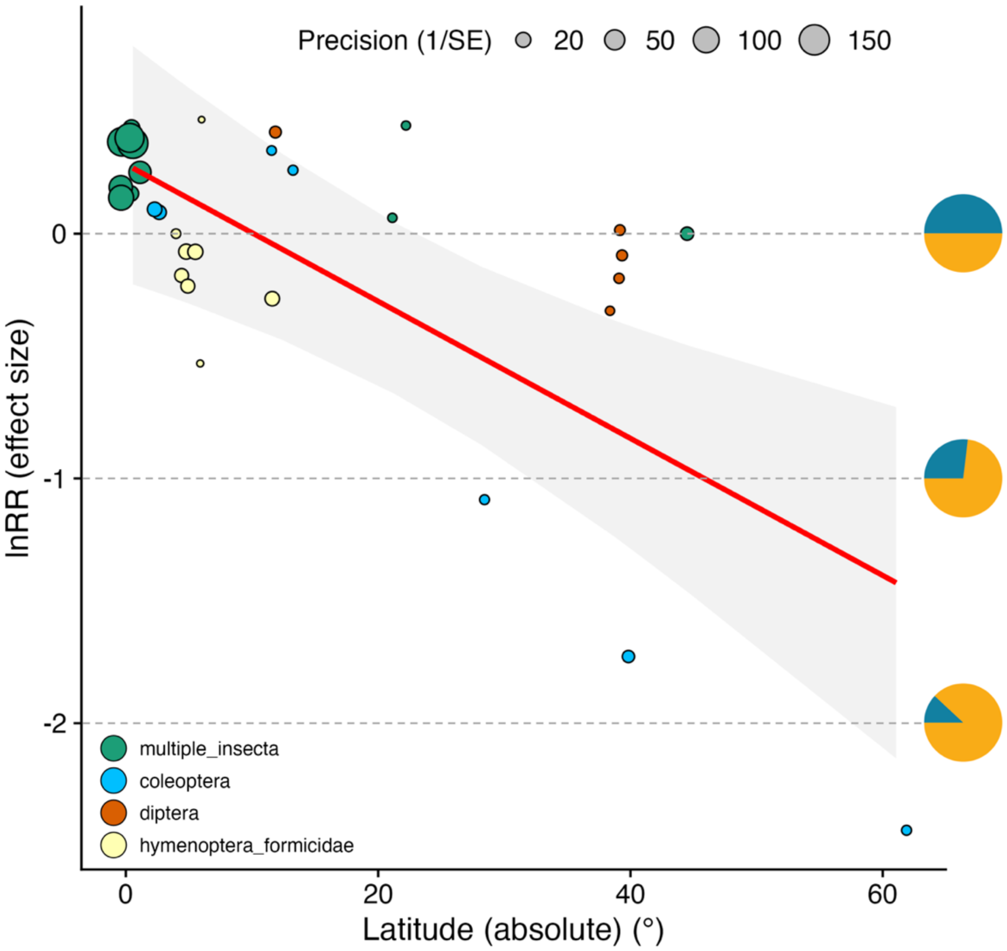

Supplementary Fig. 1. The effect of diel period on the richness of active insects in a community as a function of absolute latitude (described in main text). Plot shows the distribution of 31 effect sizes along a gradient of absolute latitude, where increasingly negative and positive effect sizes correspond to higher richness in the day and night, respectively. The colour of each dot indicates the taxonomic composition of the community. The vertical positions of pie charts correspond to effect size values, with each chart illustrating the relative proportions of taxa in a community that would be active in the day (orange) and night (teal) for a given effect size. Shaded ribbon indicates the 95% confidence interval of the mean.

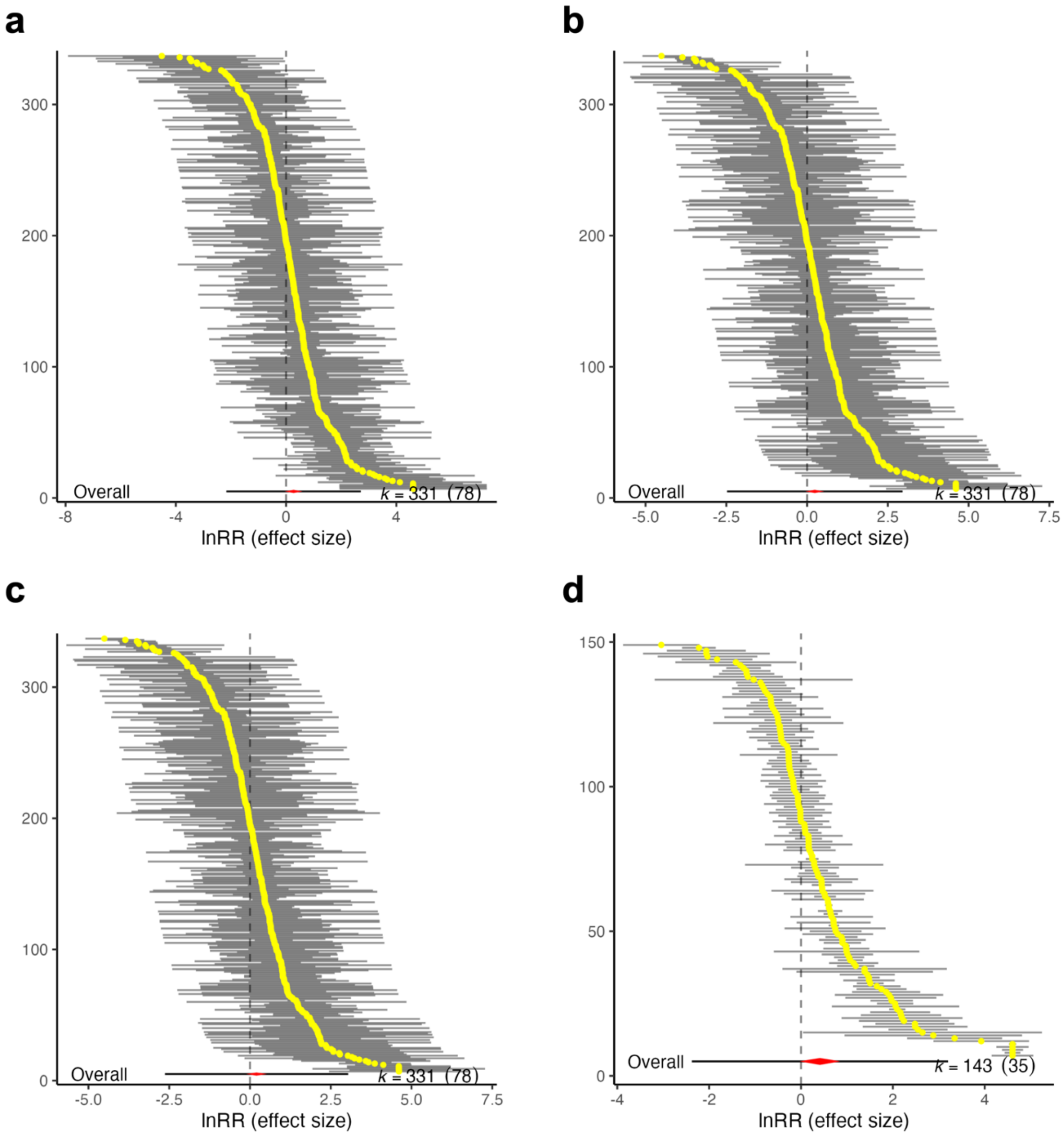

Supplementary Fig. 2. Caterpillar plots for the effect of diel period on insect abundance (see Supplementary Note 1). Increasingly negative and positive effect-size values correspond to higher insect abundance in the day and night, respectively. The overall mean effect size is centred in the red diamond at the bottom of the plot. The confidence intervals for the mean effect extend horizontally to the ends of the red diamond while the prediction intervals (black lines) extend from the red diamond. Individual plots show the results of **(a)** the meta-analysis presented in the main text; **(b)** a sensitivity analysis assuming a low correlation (rho value of 0.1) among sample variances of effect sizes obtained from the same sampling events; **(c)** a sensitivity analysis assuming a high correlation (rho value of 0.9) among sample variances of effect sizes obtained from the same sampling events; **(d)** a sensitivity analysis in which ‘complete-case analysis’ was used and the meta-analysis included only the effect sizes from 143 day–night comparisons from 35 studies which reported standard deviations.

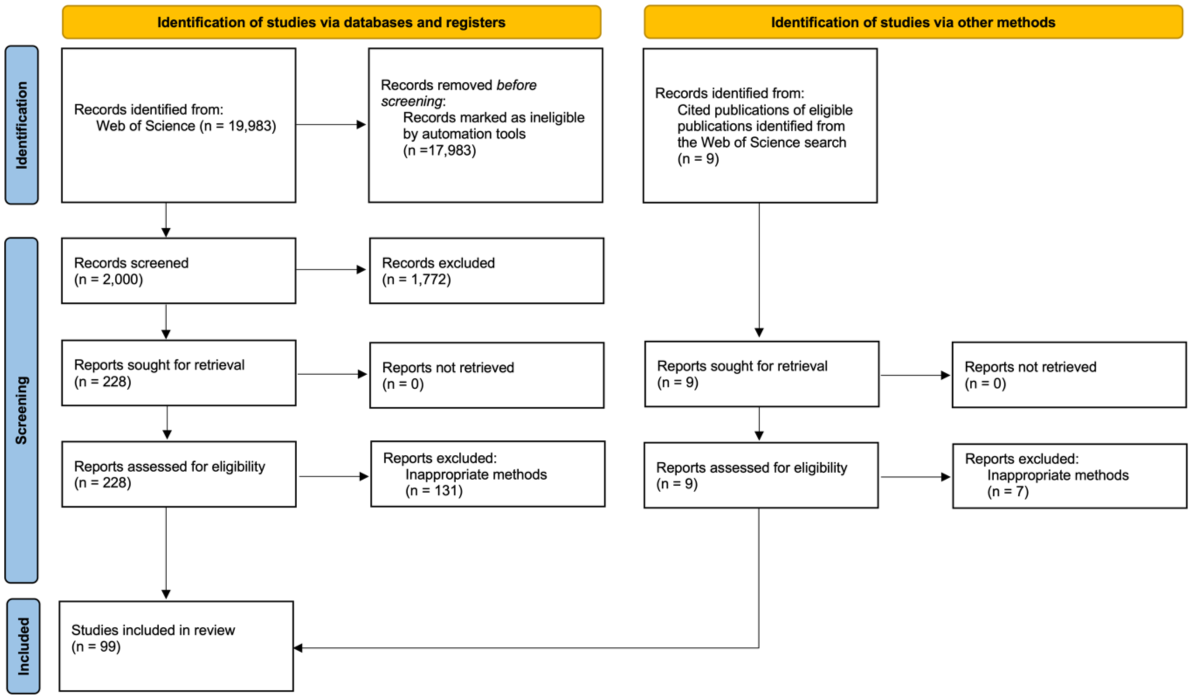

Supplementary Fig. 3. A PRISMA 2020[1] flow diagram summarising the systematic search of relevant literature used in the meta-analyses on the effects of diel period on abundance and richness in insect communities.

Supplementary Table 1. Summary details of all multi-level meta-analytical models tested for the effect of diel period on insect abundance (described in main text). All models included a publication identifier and an effect size identifier as random effects. Presented – in order of ascending AIC value – are the details of the intercept-only model which only included random effects (model 'RE.null'), 18 single-moderator models, and the full multi-moderator model (model 'RE.multimod'). AIC values were calculated from models estimated with maximum likelihood (for facilitating model comparisons), while R^2 and I^2 values were calculated from models estimated with restricted maximum likelihood (for reporting). Models positioned above the horizontal line in the table outperformed the intercept-only model based on AIC.

Model	Moderator(s)	AIC	Marginal R^2	Conditional R^2	I^2_{Total}	$I^2_{Publication}$	$I^2_{Effect\ size}$
RE.multimod	taxa + system + scale(clim.tmax) + scale(clim.prec)	1047.98	0.34	0.76	86.89	55.73	31.17
RE.taxa	taxonomic group (18 levels: apterygota, blattodea, coleoptera, dermaptera, diptera, ephemeroptera, hemiptera, hymenoptera_formicidae, hymenoptera_other, lepidoptera, multiple_insecta, neuroptera, odonata, orthoptera, plecoptera, psocoptera, thysanoptera, trichoptera)	1071.92	0.16	0.71	88.51	57.95	30.56
RE.sampling	sampling (3 levels: attraction, movement.interception, other)	1074.31	0.12	0.69	89.66	58.48	31.18
RE.habitat	habitat (6 levels: terrestrial.other, forest, grassland/savanna, aquatic.other, stream, river)	1076.10	0.24	0.65	87.17	47.10	40.07
RE.ecosystem	ecosystem (2 levels: aquatic, terrestrial)	1077.37	0.19	0.64	87.70	48.72	38.98
RE.clim.prec	precipitation, averaged across 1000 m around site during sampling months (11–838.1)	1078.22	0.02	0.58	91.36	51.67	39.69
RE.npp	net primary productivity, averaged across 1000 m radius around site (796.3–32765)	1081.00	0.06	0.72	90.61	63.51	27.10
RE.clim.tmax	temperature, maximum (1.4–33.7)	1081.50	0.06	0.65	89.17	56.46	32.71
RE.clim.tavg	temperature, average (-0.66–29.1)	1082.15	0.05	0.65	89.21	56.63	32.58
RE.clim.tmin	temperature, minimum (-3.26–24.6)	1083.03	0.05	0.65	89.27	56.85	32.43
RE.elevation	elevation (1–3821 m)	1084.03	0.05	0.65	89.25	55.93	33.32
RE.null	null (intercept only)	1088.20	0.00	0.62	89.33	55.71	33.62
RE.latitude	latitude, absolute value, in decimal degrees (0.17–63.7)	1089.09	0.02	0.64	89.56	57.09	32.46
RE.troptemp	geographic region (2 levels: tropical, temperate)	1090.00	0.00	0.63	89.48	56.44	33.05
RE.alan.max	artificial light at night value, minimum in 1000 m radius around site (0–3.46)	1090.00	0.00	0.62	89.32	55.31	34.00
RE.alan.mean	artificial light at night value, averaged across 1000 m radius around site (0–3.7)	1090.04	0.00	0.62	89.33	55.37	33.96
RE.hfp	human footprint index value, averaged across 1000 m radius around site (0–44.7)	1090.07	0.00	0.63	89.46	56.19	33.26
RE.alan.min	artificial light at night value, maximum in 1000 m radius around site (0–3.95)	1090.08	0.00	0.62	89.34	55.45	33.89
RE.clim.srad	solar radiation, averaged across 1000 m around site during sampling months (4679–27049.5)	1090.11	0.00	0.62	89.34	55.59	33.75
RE.season	season (6 levels: temperate.both, temperate.cool, temperate.warm, tropical.both, tropical.dry, tropical.wet)	1096.27	0.01	0.63	89.56	56.33	33.23

Supplementary Table 2. Summary of all multi-level meta-analytical models tested for the effect of diel period on insect richness. All models included a publication identifier and an effect size identifier as random effects. Presented – in order of ascending AIC value – are the details of the intercept-only model which only included random effects (model 'rich.RE.null') and 18 single-moderator models. AIC values were calculated for models estimated with maximum likelihood (for facilitating model comparisons), while R^2 and I^2 values were calculated for from models estimated with restricted maximum likelihood (for reporting). Models positioned above the horizontal line in the table outperformed the intercept-only model based on AIC.
Details of individual moderators are shown in Supplementary Table 1.

Model	AIC	Marginal R^2	Conditional R^2	I^2_{Total}	$I^2_{Publication}$	$I^2_{Effect\ size}$
rich.RE.latitude	17.86	0.39	0.99	99.88	97.54	2.34
rich.RE.troptemp	19.29	0.40	0.99	99.89	97.71	2,.17
rich.RE.habitat	19.80	0.42	0.99	99.87	97.42	2.45
rich.RE.season	22.50	0.45	0.98	99.86	97.03	2.83
rich.RE.ecosystem	24.24	0.22	0.99	99.92	98.45	1.48
rich.RE.sampling	24.24	0.22	0.99	99.92	98.45	1.48
rich.RE.null	25.62	0.00	0.99	99.94	98.71	1.22
rich.RE.taxa	25.66	0.25	0.99	99.93	98.48	1.45
rich.RE.alan.min	26.13	0.00	0.99	99.93	98.66	1.28
rich.RE.alan.mean	26.45	0.00	0.99	99.93	98.64	1.29
rich.RE.alan.max	26.56	0.00	0.99	99.93	98.63	1.30
rich.RE.clim.srad	26.87	0.00	0.99	99.94	98.74	1.20
rich.RE.hfp	27.21	0.00	0.99	99.94	98.66	1.28
rich.RE.elevation	27.47	0.00	0.99	99.94	98.84	1.11
rich.RE.clim.prec	27.51	0.00	0.99	99.94	98.68	1.25
rich.RE.clim.tmin	27.56	0.00	0.99	99.94	98.65	1.28
rich.RE.npp	27.62	0.00	0.99	99.94	98.63	1.31
rich.RE.clim.tmax	27.62	0.00	0.99	99.94	98.71	1.22
rich.RE.clim.tavg	27.62	0.00	0.99	99.94	98.69	1.24

Supplementary Table 3. Sensitivity tests of meta-analysis models for the effect of diel period on insect abundance (see Supplementary Note 1). Presented are the effect size estimate and 95% confidence intervals (expressed in terms of the percentage change in insect abundance from day to night samples; back-transformed from lnRR), and the heterogeneity (I^2) values for the model reported in the main text (RE.null), as well as three models adjusted for sensitivity analyses. All values were estimated from models estimated with restricted maximum likelihood.

Model	Sensitivity adjustment	Estimate (%)	CI.lwr (%)	CI.uppr (%)	I^2_{Total}	$I^2_{Publication}$	$I^2_{Effect\ size}$
RE.null	Nil (model reported in main text)	31.39	-6.31	84.26	89.33	55.71	33.62
s.rho0.1.RE.null	Assumed a rho value of 0.1 among sample variances of effect sizes obtained from the same sampling events	34.40	-0.99	82.44	88.34	54.54	33.80
s.rho0.9.RE.null	Assumed a rho value of 0.9 among sample variances of effect sizes obtained from the same sampling events	27.40	-11.61	83.62	91.18	56.16	35.02
s.cca.RE.null	Excluded observations which did not report SDs to run ‘complete-case analysis’	50.97	-3.27	136.66	99.58	74.88	24.70

Supplementary Table 4. The potential moderators of insect diel activity patterns examined in the meta-analysis and the justifications for their inclusion in the study.

Potential moderator	Description	Justification
Ecosystem realm	The ecosystem realm of the site as reported in the study. Classified under two levels: aquatic, terrestrial.	Insects inhabiting the terrestrial and aquatic realms as well as different habitats within each realm may display contrasting diel activity patterns due to variations in their interactions with the primary medium (i.e., air versus water), the other abiotic properties (e.g., habitat structure and openness, climatic factors), or the biotic properties (e.g., predation and trophic resources) present.
Habitat type	The general habitat of the site as reported in the study. Classified under six levels: terrestrial.other, forest, grassland/savanna, aquatic.other, stream, river.	
Latitude	The absolute distance of the geographic centre of the site from the equator (in decimal degrees) as reported in the study.	Multiple environmental parameters that may influence diel activity patterns such as temperature, precipitation, solar radiation vary with latitude and elevation. Latitudinal and elevational patterns have also been documented for key facets of biodiversity such as species richness, abundance, and ecological interactions[2-4].
Geographic region	The geographic region of the site. Classified under two levels: temperate, tropical. A site was classified as tropical if it was situated within the latitudinal bands of -23.4394 and 23.4394, and classified as temperate otherwise.	
Elevation	The elevation of the site (in m) as reported in the study.	
Temperature	The average, minimum, and maximum values of atmospheric temperature (°C) within a 1000 m radius around the site during the sampling month(s), obtained from WorldClim[5].	Gradients in temperature, precipitation and solar radiation may influence the diel activity patterns of insects through changing the rates of – or placing lower and upper limits on – physiological processes such as metabolism, thermal tolerance, and desiccation[6-7].
Precipitation	The value of precipitation (mm) averaged across a 1000 m radius around the site during the sampling month(s), obtained from WorldClim[5].	
Solar radiation	The value of solar radiation (kJ/m^2/day) averaged across a 1000 m radius around the site during the sampling month(s), obtained from WorldClim[5].	
Net primary productivity	The value of net primary productivity (kg C/m²) averaged across a 1000 m radius around the site during the sampling month(s), obtained from MODIS[8].	Higher primary productivity typically means more abundant food sources (such as plants for herbivorous insects or other insects for predators), which can lead to increased insect activity during periods when these resources are most accessible. In environments with low primary productivity, insects may need to be more active during certain times of the day when resources are more abundant or accessible[9].
Sampling season	The season during which sampling was performed, as reported in the study or determined based on the reported dates of sampling and the geographic location of study site. Classified under 6 levels: temperate.warm, temperate.cool, temperate.both, tropical.wet, tropical.dry, tropical.both.<br><br>Observations from studies performed in temperate localities were classified as follows: 'temperate.warm' if the study mentioned 'summer' or 'spring' or sampling was conducted during warmer months in the northern hemisphere (May–Aug) or southern hemisphere (Nov–Feb); 'temperate.cool' if the study mentioned 'autumn' or 'winter' or sampling was conducted in the other months in each respective hemisphere (cf. temperate.warm); temperate.both if sampling was conducted during both warm and cool seasons in the respective location. Observations from tropical localities were classified 'tropical.wet', 'tropical.dry' or 'tropical.both' based on reports of sampling during the wet (i.e., rainy) season, dry season, or during both seasons, respectively.	Key climatic parameters such as temperature and precipitation vary across different seasons and may influence the diel activity patterns of insects observed during a given season[10].
Sampling method	The sampling method used to observe or collect insects as reported in the study. Classified under four	Even though data from studies using clearly unsuitable sampling methods for assessing insect diel activity patterns were excluded (i.e., methods that collected

	levels: attraction, human.observation, movement.interception, other.	inactive individuals such as sweep-netting, and methods which obviously varied in collection efficiency between diel periods such as light traps), different 'suitable' sampling methods could still disproportionately sample specific diel communities due to unknown biases in their operation.
Taxonomic group	The higher taxonomic group encompassing all the individuals sampled in the study. Most groupings correspond to insect orders. Classified under 18 levels: apterygota, blattodea, coleoptera, dermaptera, diptera, ephemeroptera, hemiptera, hymenoptera_formicidae, hymenoptera_other, lepidoptera, multiple_insecta, neuroptera, odonata, orthoptera, plecoptera, psocoptera, thysanoptera, trichoptera.	Different taxonomic groups may have evolved different diel activity patterns.
Integrated human pressures	The value of the Human Footprint Index[11], an integrated measure of human pressure in the environment, averaged across a 1000 m radius around the site.	Anthropogenic disturbances such as land use, built infrastructure, traffic and noise may disproportionately impact specific diel communities[12,13].
Artificial sky luminance	The value of artificial sky luminance (mcd/m^2), averaged across 1000 m radius around the site, from Falchi et al.[14]. Note that the distances over which insects are attracted to artificial light sources have shown to be relatively small (3–50 m in a review by Boyes et al.[15]; 10–519 m estimated by Baker & Sadovy[16]).	Artificial light at night has shown to impact nocturnal insect communities[17-18].

Supplementary Note 1. Sensitivity analyses

Meta-analyses using different assumed levels of sampling error co-variance

In addition to modelling the dependency among effect sizes due to clustering (via random effects), our meta-analytic model explicitly modelled sampling error co-variances using variance–covariance matrices generated from locality-date clusters in the data[19]. To do this, we assumed a correlation (rho) value of 0.5 among sample variances of effect sizes obtained from the same sampling events. A rho value of 0 indicates no correlation and 1 a perfect correlation. While there is limited information on the specific degree of correlation among samples of insect abundance from the same sampling events, it is reasonable to assume some correlation. For instance, adverse weather conditions on the day of sampling may influence the abundance of insects sampled across different taxa, locations or habitats. As is commonly done in meta-analyses[20], we therefore conservatively assumed a rho value of 0.5. To test the sensitivity of our analyses (after Bishop & Nakagawa[21]), we re-ran the meta-analysis using separate rho values of 0.1 (low correlation) and 0.9 (high correlation) to generate the variance–covariance matrices. The results we obtained from these sensitivity analyses were qualitatively similar to those reported in the main text (Supplementary Fig. 2 and Supplementary Table 3).

Meta-analysis using 'complete-case analysis' instead of 'all cases'

In the main text, we report results of a multi-level meta-analysis for 331 day–night comparisons of abundance in insect communities from 78 studies. As data on standard deviations were not reported in 55% of studies (*n*=43), we successfully calculated values of effect size (y_i) and sampling variance (v_i) based on the average between-study coefficient of variation. Termed the 'all cases' method for estimating effect sizes, it has been shown to perform with minimal bias, regardless of the extent of missingness in the data, and has even shown to outperform conventional approaches to estimating effect sizes and sampling variances from complete data (see Nakagawa et al.[22]).

As a sensitivity analysis, we conducted a similar meta-analysis in which effect sizes and sampling variances were calculated using a conventional approach ('complete-case analysis'; Nakagawa et al.[22]). We first excluded all day–night comparisons of insect abundance in which standard deviations were not reported. Using the remaining 'complete' data, which included 143 observations from 35 studies, we then calculated each effect size based on its individual study-specific coefficient of variation. We then ran the meta-analysis using the 143 effect sizes

representing complete cases. The results of this sensitivity analysis based on ‘complete-case analysis’ were qualitatively similar to the results of the meta-analysis using the ‘all cases’ method reported in the main text (Supplementary Fig. 2 and Supplementary Table 3). Specifically, the abundance of insects was higher in the night than in the day by an average of 51% (CI: -3.3%–135.7%), and there was high heterogeneity in the effect of diel period on insect abundance both within and between publications.

Supplementary Note 2. List of studies used in the meta-analyses

1. Alencar, J., Fereira, Z. M., Lopes, C. M., Serra-Freire, N. M., De Mello, R. P., Silva, J. D. S., & Guimarães, A. É. (2011). Biodiversity and times of activity of mosquitoes (Diptera: Culicidae) in the biome of the Atlantic Forest in the State of Rio de Janeiro, Brazil. Journal of medical entomology, 48(2), 223-231.
2. Allan, J. D. (1987). Macroinvertebrate drift in a Rocky Mountain stream. Hydrobiologia, 144, 261-268.
3. Amézquita, S., & Favila, M. E. (2011). Carrion removal rates and diel activity of necrophagous beetles (Coleoptera: Scarabaeinae) in a fragmented tropical rain forest. Environmental Entomology, 40(2), 239-246.
4. Andersen, A. (1983). Species diversity and temporal distribution of ants in the semi-arid mallee region of northwestern Victoria. Australian Journal of Ecology, 8(2), 127-137.
5. Andresen, E. (2002). Dung beetles in a Central Amazonian rainforest and their ecological role as secondary seed dispersers. Ecological Entomology, 27(3), 257-270.
6. Anjos, D. V., Caserio, B., Rezende, F. T., Ribeiro, S. P., Del-Claro, K., & Fagundes, R. (2017). Extrafloral-nectaries and interspecific aggressiveness regulate day/night turnover of ant species foraging for nectar on Bionia coriacea. Austral Ecology, 42(3), 317-328.
7. Asha, G., Manoj, K., Megha, P. P., & Sinu, P. A. (2021). Spatiotemporal effects on dung beetle activities in island forests-home garden matrix in a tropical village landscape. Scientific Reports, 11(1), 17398.
8. Bailey, P. C. (1981). Insect drift in Condor Creek, Australian Capital Territory. Marine and Freshwater Research, 32(1), 111-120.
9. Basset, Y., Aberlenc, H. P., Barrios, H., Curletti, G., Bérenger, J. M., Vesco, J. P., ... & O'meara, R. (2001). Stratification and diel activity of arthropods in a lowland rainforest in Gabon. Biological Journal of the Linnean Society, 72(4), 585-607.
10. Benke, A. C., Hunter, R. J., & Parrish, F. K. (1986). Invertebrate drift dynamics in a subtropical blackwater river. Journal of the North American Benthological Society, 5(3), 173-190.
11. Bergey, E. A., & Ward, J. V. (1989). Upstream-downstream movements of aquatic invertebrates in a Rocky Mountain stream. Hydrobiologia, 185, 71-82.
12. Bogatov, V. V., & Astakhov, M. V. (2011). Under-ice drift of invertebrates in the piedmont part of Kedrovaya River (Primorskii Krai). Inland Water Biology, 4, 56-64.
13. Brewin, P. A., & Ormerod, S. J. (1994). Macroinvertebrate drift in streams of the Nepalese Himalaya. Freshwater Biology, 32(3), 573-583.
14. Briese, D. T., & Macauley, B. J. (1980). Temporal structure of an ant community in semi-arid Australia. Australian Journal of Ecology, 5(2), 121-134.

15. Butler, M. J., & Hobbs, H. H. (1982). Drift and upstream movement of invertebrates in a springbrook community ecosystem. Hydrobiologia, 89, 153-159.
16. Carval, D., Cotté, V., Resmond, R., Perrin, B., & Tixier, P. (2016). Dominance in a ground-dwelling ant community of banana agroecosystem. Ecology and Evolution, 6(23), 8617-8631.
17. Castro, D. M., Hughes, R. M., & Callisto, M. (2013). Effects of flow fluctuations on the daily and seasonal drift of invertebrates in a tropical river. In Annales de Limnologie-International Journal of Limnology (Vol. 49, No. 3, pp. 169-177). EDP Sciences.
18. Cerdá, X., Retana, J., & Cros, S. (1998). Critical thermal limits in Mediterranean ant species: trade-off between mortality risk and foraging performance. Functional Ecology, 12(1), 45-55.
19. Chatzimanolis, S., Ashe, J. S., & Hanley, R. S. (2004). Diurnal/nocturnal activity of rove beetles (Coleoptera: Staphylinidae) on Barro Colorado Island, Panama assayed by flight intercept trap. The Coleopterists Bulletin, 58(4), 569-577.
20. Chew, R. M. (1977). Some ecological characteristics of the ants of a desert-shrub community in southeastern Arizona. American Midland Naturalist, 33-49.
21. Cole, L. J., McCracken, D. I., Dennis, P., Downie, I. S., Griffin, A. L., Foster, G. N., ... & Waterhouse, T. (2002). Relationships between agricultural management and ecological groups of ground beetles (Coleoptera: Carabidae) on Scottish farmland. Agriculture, Ecosystems & Environment, 93(1-3), 323-336.
22. Costa III, J. T., & Crossley Jr, D. A. (1991). Diel patterns of canopy arthropods associated with three tree species. Environmental Entomology, 20(6), 1542-1548.
23. da Silva, P. G., Lobo, J. M., & Hernández, M. I. M. (2019). The role of habitat and daily activity patterns in explaining the diversity of mountain Neotropical dung beetle assemblages. Austral Ecology, 44(2), 300-312.
24. de Oca T, E. M., & Halffter, G. (1995). Daily and seasonal activities of a guild of the coprophagous, burrowing beetle (Coleoptera Scarabaeidae Scarabaeinae) in tropical grassland. Tropical Zoology, 8(1), 159-180.
25. Doube, B. M. (1983). The habitat preference of some bovine dung beetles (Coleoptera: Scarabaeidae) in Hluhluwe Game Reserve, South Africa. Bulletin of Entomological Research, 73(3), 357-371.
26. Dudgeon, D. (2006). The impacts of human disturbance on stream benthic invertebrates and their drift in North Sulawesi, Indonesia. Freshwater biology, 51(9), 1710-1729.
27. Feer, F., & Pincebourde, S. (2005). Diel flight activity and ecological segregation within an assemblage of tropical forest dung and carrion beetles. Journal of Tropical Ecology, 21(1), 21-30.

28. Ferrington, L. C. (1984). Drift dynamics of Chironomidae larvae: I. Preliminary results and discussion of importance of mesh size and level of taxonomic identification in resolving Chironomidae diel drift patterns. Hydrobiologia, 114, 215-227.
29. Forattini, O. P., Alves, A. D. C., Natal, D., & Santos, J. L. F. (1986). Observações sobre atividade de mosquitos Culicidae em mata primitiva da encosta no Vale do Ribeira, São Paulo, Brasil. Revista de Saúde Pública, 20, 1-20.
30. Frizzi, F., Tucci, L., Ottonetti, L., Masoni, A., & Santini, G. (2021). Day-night and inter-habitat variations in ant assemblages in a mosaic agroforestry landscape. Land, 10(2), 179.
31. Giroux, F., Ovidio, M., Phillippart, J. C., & Baras, E. (2000). Relationship between the drift of macroinvertebrates and the activity of brown trout in a small stream. Journal of Fish Biology, 56(5), 1248-1257.
32. Grevé, M. E., Houadria, M., Andersen, A. N., & Menzel, F. (2019). Niche differentiation in rainforest ant communities across three continents. Ecology and Evolution, 9(15), 8601-8615.
33. Gryuntal, S. (2008). Daily activity of carabid beetles (Coleoptera: Carabidae) in the forests of various geographical zones in the East European (Russian) Plain. Russian Entomological Journal, 17, 359-365.
34. Guareschi, S., Laini, A., Fenoglio, S., Paveto, M., & Bo, T. (2016). Change does not happen overnight: a case study on stream macroinvertebrates. Knowledge and Management of Aquatic Ecosystems, (417), 21.
35. Guevara, J., & Avilés, L. (2013). Community-wide body size differences between nocturnal and diurnal insects. Ecology, 94(2), 537-543.
36. Guimarães, A. É., Gentile, C., Lopes, C. M., & Mello, R. P. D. (2000). Ecology of mosquitoes (Diptera: Culicidae) in areas of Serra do Mar State Park, State of São Paulo, Brazil. III-daily biting rhythms and lunar cycle influence. Memórias do Instituto Oswaldo Cruz, 95, 753-760.
37. Hampton, S. E., & Friedenberg, N. A. (2002). Nocturnal increases in the use of near-surface water by pond animals. Hydrobiologia, 477, 171-179.
38. Hardwick, R. A., Cooper, P. D., Cranston, P. S., Humphrey, C. L., & Dostine, P. L. (1995). Spatial and temporal distribution patterns of drifting pupal exuviae of Chironomidae (Diptera) in streams of tropical northern Australia. Freshwater Biology, 34(3), 569-578.
39. Hashimoto, Y., Morimoto, Y., Widodo, E. S., Mohamed, M., & Fellowes, J. R. (2010). Vertical habitat use and foraging activities of arboreal and ground ants (Hymenoptera: Formicidae) in a Bornean tropical rainforest. Sociobiology, 56(2), 435.
40. Heatwole, H. (1991). The ant assemblage of a sand-dune desert in the United Arab Emirates. Journal of Arid Environments, 21(1), 71-79.

41. Heatwole, H., Trémont, S., & Broese, E. (2013). Point-diversity, a critical tool for assessing dynamics of guilds of scavenging ants (Hymenoptera: Formicidae): an example from a eucalypt woodland. Systematics and Biodiversity, 11(2), 149-180.
42. Hernández, M. I. M. (2002). The night and day of dung beetles (Coleoptera, Scarabaeidae) in the Serra do Japi, Brazil: elytra colour related to daily activity. Revista brasileira de Entomologia, 46, 597-600.
43. Hieber, M., Robinson, C. T., & Uehlinger, U. (2003). Seasonal and diel patterns of invertebrate drift in different alpine stream types. Freshwater Biology, 48(6), 1078-1092.
44. Hill, C. J. (1996). Habitat specificity and food preferences of an assemblage of tropical Australian dung beetles. Journal of Tropical Ecology, 12(4), 449-460.
45. Horgan, F. G. (2002). Shady field boundaries and the colonisation of dung by coprophagous beetles in Central American pastures. Agriculture, ecosystems & environment, 91(1-3), 25-36.
46. Hossaert-McKey, M., Orivel, J., Labeyrie, E., Pascal, L., Delabie, J., & Dejean, A. (2001). Differential associations with ants of three cooccurring extrafloral nectary-bearing plants. Ecoscience, 8(3), 325-335.
47. Jacobsen, D., & Bojsen, B. (2002). Macroinvertebrate drift in Amazon streams in relation to riparian forest cover and fish fauna. Archiv fur Hydrobiologie, 155(2), 177-197.
48. Johnson, C. G., Crosskey, R. W., & Davies, J. B. (1982). Species composition and cyclical changes in numbers of savanna blackflies (Diptera: Simuliidae) caught by suction traps in the Onchocerciasis Control Programme area of West Africa. Bulletin of Entomological Research, 72(1), 39-63.
49. Kamiński, M. J., Byk, A., & Tykarski, P. (2015). Seasonal and diel activity of dung beetles (Coleoptera: Scarabaeoidea) attracted to European bison dung in Białowieża Primeval Forest, Poland. The Coleopterists Bulletin, 69(1), 83-90.
50. Kaspari, M., & Weiser, M. D. (2000). Ant activity along moisture gradients in a neotropical forest 1. Biotropica, 32(4a), 703-711.
51. Kirmse, S., & Ratcliffe, B. C. (2019). Composition and host-use patterns of a scarab beetle (Coleoptera: Scarabaeidae) community inhabiting the canopy of a lowland tropical rainforest in southern Venezuela. The Coleopterists Bulletin, 73(1), 149-167.
52. Knop, E., Gerpe, C., Ryser, R., Hofmann, F., Menz, M. H., Trösch, S., ... & Fontaine, C. (2018). Rush hours in flower visitors over a day–night cycle. Insect Conservation and Diversity, 11(3), 267-275.
53. Kocarek, P. (2002). Diel activity patterns of carrion-visiting Coleoptera studied by time-sorting pitfall traps. Biologia Bratislava, 57(2), 199-212.
54. Koskela, H. (1979). Patterns of diel flight activity in dung-inhabiting beetles: an ecological analysis. Oikos, 419-439.

55. Krell-Westerwalbesloh, S., Krell, F. T., & Eduard Linsenmair, K. (2004). Diel separation of Afrotropical dung beetle guilds—avoiding competition and neglecting resources (Coleoptera: Scarabaeoidea). Journal of Natural History, 38(17), 2225-2249.
56. Krell, F. T., Krell-Westerwalbesloh, S., Weiß, I., Eggleton, P., & Linsenmair, K. E. (2003). Spatial separation of Afrotropical dung beetle guilds: a trade-off between competitive superiority and energetic constraints (Coleoptera: Scarabaeidae). Ecography, 26(2), 210-222.
57. Lieberman, S., & Dock, C. F. (1982). Analysis of the leaf litter arthropod fauna of a lowland tropical evergreen forest site (La Selva, Costa Rica). Revista de Biologia Tropical, 30(1), 27-34.
58. Lindsey, P. A., & Skinner, J. D. (2001). Ant composition and activity patterns as determined by pitfall trapping and other methods in three habitats in the semi-arid Karoo. Journal of Arid Environments, 48(4), 551-568.
59. Liu, X., Wang, Z., Huang, C., Li, M., Bibi, F., Zhou, S., & Nakamura, A. (2020). Ant assemblage composition explains high predation pressure on artificial caterpillars during early night. Ecological Entomology, 45(3), 547-554.
60. Lobo, J. M., & Cuesta, E. (2021). Seasonal variation in the diel activity of a dung beetle assemblage. PeerJ, 9, e11786.
61. Lobón-Cerviá, J., Rezende, C. F., & Castellanos, C. (2012). High species diversity and low density typify drift and benthos composition in Neotropical streams. Fundamental and Applied Limnology-Archiv furHydrobiologie, 181(2), 129.
62. Luna, P., Peñaloza-Arellanes, Y., Castillo-Meza, A. L., García-Chávez, J. H., & Dáttilo, W. (2018). Beta diversity of ant-plant interactions over day-night periods and plant physiognomies in a semiarid environment. Journal of Arid Environments, 156, 69-76.
63. Majzlan, O., & Bayartogtokh, B. (1989). Structure and dynamics of epigeon of the semidesert and desert zone of Mongolian People's Republic. Biológia (Bratislava), 44(6), 533-540.
64. Marchiori, C. H., Lussari, M. A., Rosa, D. C., & Penteado-Dias, A. M. (2007). Parasitoid Hymenoptera collected during the diurnal and nocturnal periods in Itumbiara, Goiás. Brazilian Journal of Biology, 67, 581-582.
65. Marques, G. D. V., & Del-Claro, K. (2006). The ant fauna in a Cerrado area: the influence of vegetation structure and seasonality (Hymenoptera: Formicidae). Sociobiology, 47(1), 235-252.
66. Mathooko, J. M., & Mavuti, K. M. (1994). Diel dynamics of organic drift transport in a second-order, high-altitude river in Kenya. African Journal of Ecology, 32(3), 259-263.
67. Medina, A. M., & Lopes, P. P. (2014). Resource utilization and temporal segregation of Scarabaeinae (Coleoptera, Scarabaeidae) community in a Caatinga fragment. Neotropical entomology, 43, 127-133.

68. Móra, A., Dévai, G., Tóthmérész, B., & Csépes, E. (2006). Short-time changes in composition of chironomid assemblages at a cross-section of the River Tisza. Internationale Vereinigung für theoretische und angewandte Limnologie: Verhandlungen, 29(4), 2099-2102.
69. Newman, D. L., & Funk, R. C. (1984). Drift of riffle beetles (Coleoptera: Elmidae) in a small Illinois stream. The Great Lakes Entomologist, 17(4), 5.
70. O'Leary, P., Lake, P. S., Marchant, R., & Doeg, T. J. (1992). Macroinvertebrate activity in the water column of backwaters in an upland stream in Victoria. Marine and Freshwater Research, 43(6), 1403-1407.
71. Obi, A., & Conner, J. V. (1986). Spring and summer macroinvertebrate drift in the Lower Mississippi River, Louisiana. Hydrobiologia, 139, 167-175.
72. Oliveira, D. L., & Vasconcelos, S. D. (2018). Diversity, daily flight activity and temporal occurrence of necrophagous Diptera associated with decomposing carcasses in a semi-arid environment. Neotropical entomology, 47, 470-477.
73. Pardo-Locarno, L. C. (2007). Dung beetles (Coleoptera-Scarabaeidae) from lloró, department of Chocó, Colombia. Boletín Científico. Centro de Museos. Museo de Historia Natural, 11(1), 377-388.
74. Pringle, C. M., & Ramírez, A. (1998). Use of both benthic and drift sampling techniques to assess tropical stream invertebrate communities along an altitudinal gradient, Costa Rica. Freshwater Biology, 39(2), 359-373.
75. Ramírez, A., & Pringle, C. M. (1998). Invertebrate drift and benthic community dynamics in a lowland neotropical stream, Costa Rica. Hydrobiologia, 386, 19-26.
76. Ramírez, A., & Pringle, C. M. (2001). Spatial and temporal patterns of invertebrate drift in streams draining a Neotropical landscape. Freshwater Biology, 46(1), 47-62.
77. Ríos-Touma, B., Prat, N., & Encalada, A. C. (2012). Invertebrate drift and colonization processes in a tropical Andean stream. Aquatic Biology, 14(3), 233-246.
78. Sagar, P. M., & Glova, G. J. (1992). Diel changes in the abundance and size composition of invertebrate drift in five rivers in South Island, New Zealand. New Zealand journal of marine and freshwater research, 26(1), 103-114.
79. Saltveit, S. J., Haug, I., & Brittain, J. E. (2001). Invertebrate drift in a glacial river and its non-glacial tributary. Freshwater Biology, 46(12), 1777-1789.
80. Sánchez-Hernández, G., Gómez, B., Rodríguez-López, M. E., Dávila-Sánchez, R. A., & Chamé-Vázquez, E. R. (2021). Variation in dung beetle (Coleoptera: Scarabaeidae: Scarabaeinae) assemblages in a tropical forest remnant from a Mexican National Park. Papéis Avulsos de Zoologia, 61.

81. Sands, B., Mgidiswa, N., Curson, S., Nyamukondiwa, C., & Wall, R. (2022). Dung beetle community assemblages in a southern African landscape: niche overlap between domestic and wild herbivore dung. Bulletin of Entomological Research, 112(1), 131-142.
82. Schreiber, E. S. G. (1995). Long-term patterns of invertebrate stream drift in an Australian temperate stream. Freshwater Biology, 33(1), 13-25.
83. Soares, T. F., & Vasconcelos, S. D. (2016). Diurnal and nocturnal flight activity of blow flies (Diptera: Calliphoridae) in a rainforest fragment in Brazil: implications for the colonization of homicide victims. Journal of forensic sciences, 61(6), 1571-1577.
84. Springate, N. D., & Basset, Y. (1996). Diel activity of arboreal arthropods associated with Papua New Guinean trees. Journal of Natural History, 30(1), 101-112.
85. Stapp, P. (1997). Microhabitat use and community structure of darkling beetles (Coleoptera: Tenebrionidae) in shortgrass prairie: effects of season shrub and soil type. American Midland Naturalist, 298-311.
86. Stringer, I. A., & Meyer-Rochow, V. B. (1997). Flight activity of insects within a Jamaican cave: in search of the zeitgeber. Invertebrate Biology, 348-354.
87. Sullivan, G. T., Ozman-Sullivan, S. K., Lumaret, J. P., Bourne, A., Zeybekoglu, U., Zalucki, M. P., & Baxter, G. (2017). How guilds build success; aspects of temporal resource partitioning in a warm, temperate climate assemblage of dung beetles (Coleoptera: Scarabaeidae). Environmental entomology, 46(5), 1060-1069.
88. Tanaka, H. O., Yamane, S., & Itioka, T. (2010). Within-tree distribution of nest sites and foraging areas of ants on canopy trees in a tropical rainforest in Borneo. Population Ecology, 52, 147-157.
89. Tilley, L. J. (1989). Diel drift of Chironomidae larvae in a pristine Idaho mountain stream. Hydrobiologia, 174, 133-149.
90. Torres, J. A. (1984). Niches and coexistence of ant communities in Puerto Rico: repeated patterns. Biotropica, 284-295.
91. Turcotte, P., & Harper, P. P. (1982). Drift patterns in a high Andean stream. Hydrobiologia, 89, 141-151.
92. Ubero-Pascal, N., Torralva, M., Oliva-Paterna, F., & Malo, J. (2000). Seasonal and diel periodicity of the drift of pupal exuviae of chironomid (Diptera) in the Mundo River (SE Spain). Arch. Hydrobiol, 147(2), 161-170.
93. Van de Meutter, F., Stoks, R., & De Meester, L. (2006). Lotic dispersal of lentic macroinvertebrates. Ecography, 29(2), 223-230.
94. Viljanen, H., Wirta, H., Montreuil, O., Rahagalala, P., Johnson, S., & Hanski, I. (2010). Structure of local communities of endemic dung beetles in Madagascar. Journal of Tropical Ecology, 26(5), 481-496.

95. Williams, G. (1959). The seasonal and diurnal activity of the fauna sampled by pitfall traps in different habitats. The Journal of Animal Ecology, 1-13.
96. Willmer, P. G. (1982). Hygrothermal determinants of insect activity patterns: the Diptera of water-lily leaves. Ecological Entomology, 7(2), 221-231.
97. Yamane, S., Itino, T., & Nona, A. R. (1996). Ground ant fauna in a Bornean dipterocarp forest. Raffles Bulletin of Zoology, 44(1), 253-262.
98. Young, A. M. (1986). Distribution and abundance of Diptera in flypaper traps at Theobroma cacao L.(Sterculiaceae) flowers in Costa Rican cacao plantations. Journal of the Kansas Entomological Society, 580-587.
99. Yusah, K. M., Foster, W. A., Reynolds, G., & Fayle, T. M. (2018). Ant mosaics in Bornean primary rain forest high canopy depend on spatial scale, time of day, and sampling method. PeerJ, 6, e4231.

Supplementary References

1. Page, M. J., McKenzie, J. E, Bossuyt, P. M., Boutron, I., Hoffmann, T. C., Mulrow, C. D., et al. The PRISMA 2020 statement: an updated guideline for reporting systematic reviews. *Br. Med. J.* **372**, 71 (2021).
2. Janzen, D. H. Why mountain passes are higher in the tropics. *Am. Nat.* **101**, 233-249 (1967).
3. Willig, M. R., Kaufman, D. M., & Stevens, R. D. Latitudinal gradients of biodiversity: pattern, process, scale, and synthesis. *Annu. Rev. Ecol. Evol. Syst.* **34**, 273-309 (2003).
4. Roslin, T., Hardwick, B., Novotny, V., Petry, W. K., Andrew, N. R., Asmus, A. et al. Higher predation risk for insect prey at low latitudes and elevations. *Science* **356**, 742-744 (2017).
5. Fick, S. E. & Hijmans, R. J. WorldClim 2: new 1-km spatial resolution climate surfaces for global land areas. *Int. J. Climatol.* **37**, 4302-4315 (2017).
6. Addo-Bediako, A., Chown, S. L. & Gaston, K. J. Thermal tolerance, climatic variability and latitude. *Proc. Royal Soc. B.* **267**, 739-745 (2000).
7. Battisti, A., Marini, L., Pitacco, A., & Larsson, S. Solar radiation directly affects larval performance of a forest insect. *Ecol. Entomol.* **38**, 553-559 (2013).
8. Justice, C. O., Townshend, J. R. G., Vermote, E. F., Masuoka, E., Wolfe, R. E., Saleous, N. et al. An overview of MODIS Land data processing and product status. *Remote Sens. Environ.* **83**, 3-15 (2002).
9. Janzen, D. H. Food webs: who eats what, why, how, and with what effects in a tropical forest?. In: *Tropical rain forest ecosystems* (ed. F. B. I. Golley) (Elsevier, Amsterdam, 1983).
10. Wolda, H. Insect seasonality: why?. *Annu. Rev. Ecol. Evol. Syst.* **19**, 1-18 (1988).
11. Venter, O., Sanderson, E. W., Magrach, A., Allan, J. R., Beher, J., Jones, K. R. et al. Global terrestrial Human Footprint maps for 1993 and 2009. *Sci. Data* **3**, 1-10 (2016).
12. Gaynor, K. M., Hojnowski, C. E., Carter, N. H., & Brashares, J. S. The influence of human disturbance on wildlife nocturnality. *Science* **360**, 1232-1235 (2018).
13. Schowalter, T. D. Insect responses to major landscape-level disturbance. *Annu. Rev. Entomol.* **57**, 1-20 (2012).
14. Falchi, F., Cinzano, P., Duriscoe, D., Kyba, C. C., Elvidge, C. D., Baugh, K. ... & Furgoni, R. The new world atlas of artificial night sky brightness. *Sci. Adv.* **2**, e1600377 (2016).

15. Boyes, D. H., Evans, D. M., Fox, R., Parsons, M. S. & Pocock, M. J. Is light pollution driving moth population declines? A review of causal mechanisms across the life cycle. *Insect Conserv. Divers.* **14**, 167-187 (2021).

16. Baker, R. R. & Sadovy, Y. The distance and nature of the light-trap response of moths. *Nature* **276**, 818-821 (1978).

17. Owens, A. C., & Lewis, S. M. The impact of artificial light at night on nocturnal insects: a review and synthesis. *Ecol. Evol.* **8**, 11337-11358 (2018).

18. Boyes, D. H., Evans, D. M., Fox, R., Parsons, M. S., & Pocock, M. J. Street lighting has detrimental impacts on local insect populations. *Sci. Adv.* **7**, eabi8322 (2021).

19. Noble, D. W., Lagisz, M., O'dea, R. E., & Nakagawa, S. Nonindependence and sensitivity analyses in ecological and evolutionary meta-analyses. *Mol. Ecol.* **26**, 2410-2425 (2017).

20. Dougherty, L. R., Skirrow, M. J., Jennions, M. D. & Simmons, L. W. Male alternative reproductive tactics and sperm competition: A meta-analysis. *Biol. Rev.* **97**, 1365-1388 (2022).

21. Bishop, J. & Nakagawa, S. Quantifying crop pollinator dependence and its heterogeneity using multi-level meta-analysis. *J. Appl. Ecol.* **58**, 1030-1042 (2021).

22. Nakagawa, S., Noble, D. W., Lagisz, M., Spake, R., Viechtbauer, W. & Senior, A. M. A robust and readily implementable method for the meta-analysis of response ratios with and without missing standard deviations. *Ecol. Lett.* **26**, 232-244 (2023).
