# Supplementary material for: Global meta-analysis reveals overall higher nocturnal than diurnal activity in insect communities
Source: Nat Commun. 2024 Apr 15;15:3236. doi: 10.1038/s41467-024-47645-2 (PMC11018786; doi:10.1038/s41467-024-47645-2)
Supplement: Supplementary file 3 — Reporting Summary [file 41467_2024_47645_MOESM3_ESM.pdf]

nature portfolio

Corresponding author(s): Mark K. L. Wong

Last updated by author(s): Mar 22, 2024

Reporting Summary

Nature Portfolio wishes to improve the reproducibility of the work that we publish. This form provides structure for consistency and transparency in reporting. For further information on Nature Portfolio policies, see our Editorial Policies and the Editorial Policy Checklist.

Statistics

For all statistical analyses, confirm that the following items are present in the figure legend, table legend, main text, or Methods section.

n/a	Confirmed	
☐	☒	The exact sample size (*n*) for each experimental group/condition, given as a discrete number and unit of measurement
☐	☒	A statement on whether measurements were taken from distinct samples or whether the same sample was measured repeatedly
☐	☒	The statistical test(s) used AND whether they are one- or two-sided<br>*Only common tests should be described solely by name; describe more complex techniques in the Methods section.*
☐	☒	A description of all covariates tested
☒	☐	A description of any assumptions or corrections, such as tests of normality and adjustment for multiple comparisons
☐	☒	A full description of the statistical parameters including central tendency (e.g. means) or other basic estimates (e.g. regression coefficient) AND variation (e.g. standard deviation) or associated estimates of uncertainty (e.g. confidence intervals)
☐	☒	For null hypothesis testing, the test statistic (e.g. *F*, *t*, *r*) with confidence intervals, effect sizes, degrees of freedom and *P* value noted<br>*Give P values as exact values whenever suitable.*
☒	☐	For Bayesian analysis, information on the choice of priors and Markov chain Monte Carlo settings
☐	☒	For hierarchical and complex designs, identification of the appropriate level for tests and full reporting of outcomes
☐	☒	Estimates of effect sizes (e.g. Cohen's *d*, Pearson's *r*), indicating how they were calculated

Our web collection on statistics for biologists contains articles on many of the points above.

Software and code

Policy information about availability of computer code

Data collection	No software was used for the data collection in this study.
Data analysis	Data analysis was performed using the metafor package and visualized with the orchaRd package in R software version 4.3.1.

For manuscripts utilizing custom algorithms or software that are central to the research but not yet described in published literature, software must be made available to editors and reviewers. We strongly encourage code deposition in a community repository (e.g. GitHub). See the Nature Portfolio guidelines for submitting code & software for further information.

Data

Policy information about availability of data

All manuscripts must include a data availability statement. This statement should provide the following information, where applicable:

- Accession codes, unique identifiers, or web links for publicly available datasets
- A description of any restrictions on data availability
- For clinical datasets or third party data, please ensure that the statement adheres to our policy

All data generated in this study and all code supporting the findings of this study have been deposited at Figshare with the identifier https://doi.org/10.6084/m9.figshare.24164652

Research involving human participants, their data, or biological material

Policy information about studies with human participants or human data. See also policy information about sex, gender (identity/presentation), and sexual orientation and race, ethnicity and racism.

Reporting on sex and gender	N/A
Reporting on race, ethnicity, or other socially relevant groupings	N/A
Population characteristics	N/A
Recruitment	N/A
Ethics oversight	N/A

Note that full information on the approval of the study protocol must also be provided in the manuscript.

Field-specific reporting

Please select the one below that is the best fit for your research. If you are not sure, read the appropriate sections before making your selection.

☐ Life sciences ☐ Behavioural & social sciences ☒ Ecological, evolutionary & environmental sciences

For a reference copy of the document with all sections, see nature.com/documents/nr-reporting-summary-flat.pdf

Ecological, evolutionary & environmental sciences study design

All studies must disclose on these points even when the disclosure is negative.

Study description	To investigate the extent and the moderators of diel variation in abundance and richness in insect communities, we conducted a meta-analysis following a systematic search of the literature.
Research sample	We identified 99 publications from the peer-reviewed literature which systematically sampled insect communities in day and night periods with appropriate methods for detecting diel patterns in insect activity.
Sampling strategy	We performed a literature search in all Web of Science (WoS) databases on 28th April 2022 for studies that sampled insect communities across the diel cycle. The search terms used were '(insect) AND (community OR communities) AND (activity OR diel OR nocturnal OR diurnal OR night OR day)'. We sorted the results by relevance and screened the abstracts of the first 2000 results to identify relevant publications (beyond the 1350th result, zero to two relevant publications were identified out of every 50 results, while no relevant publications were identified beyond the 1850th result). We only included studies that systematically sampled insect communities with identical collection methods in day and night periods, which were defined within studies and consistently referred to as the period after sunrise and before sunset, and the period after sunset and before sunrise, respectively. We included studies that used collection methods that collected active individuals, such as movement-based interception traps (e.g., pitfall traps, sticky traps, malaise traps, drift nets) and some attraction-based traps (e.g., dung-baited pitfall traps). We excluded studies that used methods which could potentially collect inactive individuals (e.g., sweep-netting, beating) as well as methods for which collection efficiency or attractiveness was influenced by environmental changes across the diel cycle, such as light traps.
Data collection	Data were extracted from publications using reported effect sizes in the main text, figures and supplementary data files. All data collection was performed by the first author (MKLW) using a computer and Microsoft Excel software for record keeping.
Timing and spatial scale	The studies were published between 1959 and 2022. The spatial scale was global; the sampled locations across all studies spanned all continents except Antarctica.
Data exclusions	Studies were excluded if they did not meet the data selection criteria reported above. No further exclusion criteria were applied.
Reproducibility	The study was based on existing observational data from studies on insect communities in the literature; no experiments were carried out.
Randomization	Randomization was not relevant to this meta-analytical study because meta-analysis involves the aggregation and analysis of existing data from multiple primary studies, whereas randomization is used in ndividual primary studies to minimize bias and ensure that treatment groups are comparable.
Blinding	Blinding was not relevant to the study as it was an observational study; no experiments were carried out.

Did the study involve field work? ☐ Yes ☒ No

Reporting for specific materials, systems and methods

We require information from authors about some types of materials, experimental systems and methods used in many studies. Here, indicate whether each material, system or method listed is relevant to your study. If you are not sure if a list item applies to your research, read the appropriate section before selecting a response.

Materials & experimental systems

n/a	Involved in the study
☒	☐ Antibodies
☒	☐ Eukaryotic cell lines
☒	☐ Palaeontology and archaeology
☒	☐ Animals and other organisms
☒	☐ Clinical data
☒	☐ Dual use research of concern
☒	☐ Plants

Methods

n/a	Involved in the study
☒	☐ ChIP-seq
☒	☐ Flow cytometry
☒	☐ MRI-based neuroimaging

Plants

Seed stocks	N/A
Novel plant genotypes	N/A
Authentication	N/A
